# Role of the *Vibrio cholerae* Matrix Protein Bap1 in Cross-Resistance to Antimicrobial Peptides

**DOI:** 10.1371/journal.ppat.1003620

**Published:** 2013-10-03

**Authors:** Marylise Duperthuy, Annika E. Sjöström, Dharmesh Sabharwal, Fatemeh Damghani, Bernt Eric Uhlin, Sun Nyunt Wai

**Affiliations:** Department of Molecular Biology, The Laboratory for Molecular Infection Medicine Sweden (MIMS), Umeå University, Umeå, Sweden; Yale University School of Medicine, United States of America

## Abstract

Outer membrane vesicles (OMVs) that are released from Gram-negative pathogenic bacteria can serve as vehicles for the translocation of effectors involved in infectious processes. In this study we have investigated the role of OMVs of the *Vibrio cholerae* O1 El Tor A1552 strain in resistance to antimicrobial peptides (AMPs). To assess this potential role, we grew *V. cholerae* with sub-lethal concentrations of Polymyxin B (PmB) or the AMP LL-37 and analyzed the OMVs produced and their effects on AMP resistance. Our results show that growing *V. cholerae* in the presence of AMPs modifies the protein content of the OMVs. In the presence of PmB, bacteria release OMVs that are larger in size and contain a biofilm-associated extracellular matrix protein (Bap1). We demonstrated that Bap1 binds to the OmpT porin on the OMVs through the LDV domain of OmpT. In addition, OMVs from cultures incubated in presence of PmB also provide better protection for *V. cholerae* against LL-37 compared to OMVs from *V. cholerae* cultures grown without AMPs or in presence of LL-37. Using a *bap1* mutant we showed that cross-resistance between PmB and LL-37 involved the Bap1 protein, whereby Bap1 on OMVs traps LL-37 with no subsequent degradation of the AMP.

## Author Summary

Cholera is an epidemic disease that has killed millions of people and continues to be a major health problem worldwide. The bacterium *V. cholerae*, the causative agent of cholera, is highly resistant to antimicrobial peptides, which are important effectors of human innate immunity. Moreover, it is well-established that different antimicrobial peptides are able to work in synergy against bacteria. Currently, little is known about the mechanisms underlying the resistance of bacteria toward synergistic effects of antimicrobial peptides. For the first time, we provide a mechanistic explanation for cross-resistance between two antimicrobial peptides: PmB and LL-37. We report that bacteria incubated with PmB produce OMVs containing high levels of the Bap1 protein. We also decipher the mechanism by which Bap1 binds to OMVs isolated from *V. cholerae* incubated in presence of PmB. Finally, our data demonstrated that Bap1 protein associated with OMVs is able to trap LL-37, thereby increasing the minimum inhibitory concentration of LL-37 against *V. cholerae* when the bacteria were grown with a sub-lethal concentration of PmB and therefore produced abundant Bap1.

## Introduction

*V. cholerae* is the causative agent of the disease cholera, which remains a significant public health problem, causing large numbers of infections and deaths annually in the world [1]. During the infection, *V. cholerae* colonizes the surface of the small intestine where it secretes virulence factors [2], [3].

Gram-negative bacteria constitutively release lipid bilayer vesicles during normal growth. These outer membrane vesicles (OMVs) range in size from 20–200 nm in diameter. As the vesicles are extruded from the surface of the bacterial cells, some underlying periplasmic components become entrapped inside the vesicles [4], [5]. OMVs possess outer membrane proteins, lipopolysaccharide (LPS), phospholipids, and some periplasmic constituents. They have been suggested to play diverse roles in bacterial pathogenesis, including involvement in bacterial communication through OMV-associated signaling molecules, as virulence factors, and in genetic transformation [6], [7], [8], [9], [10], [11], [12].

Antimicrobial peptides (AMPs) contribute to the innate human defense against bacterial infections, as well as in the defense employed by some bacteria against predators [13]. The intestinal epithelium is the site of synthesis of many AMPs, including defensins and cathelicidins such as LL-37, whose expression can be constitutive or induced by microorganisms [14]. Similarly, AMPs stored in Paneth cells are delivered to the lumen of the small intestine in response to bacterial stimulation [15]. Sub-inhibitory concentrations of AMPs are commonly encountered by pathogens on the epithelial surfaces, or in the environment [16]. The most common mechanism of action of AMPs is alteration of bacterial membrane permeability, by pore formation and/or lipid modifications to alter the charge of the outer membrane [17]. Resistance to AMPs is now recognized as an important virulence phenotype in many human pathogenic bacteria. Gram-negative bacteria have developed a wide range of mechanisms to overcome AMPs, such as production of proteases that degrade the peptides, production of secretory proteins that bind the AMP before it reaches its target, efflux systems that actively extrude AMPs if they access the bacterial cytoplasm, modification of the bacterial envelope in order to reduce its net anionic charge and subsequently to decrease the affinity of the cationic AMP for the outer membrane, and regulation of host AMP production [18].

In this study we examined the potential role for OMVs in resistance to AMPs of *V. cholerae* El Tor O1 strain A1552. To address this question, we isolated OMVs from *V. cholerae* cultures grown in presence of AMPs (PmB and LL-37) and compared their OMV protein profiles and AMP resistance with those of OMVs from cultures grown without AMPs. Our data suggest that OMVs from *V. cholerae* are involved in AMP cross-resistance. This cross-resistance is mediated by a biofilm-associated extracellular matrix protein (Bap1) associated with OMVs of *V. cholerae* grown with PmB.

## Results

### Effects of AMPs on OMV production from *V. cholerae*

**Figure 1:**
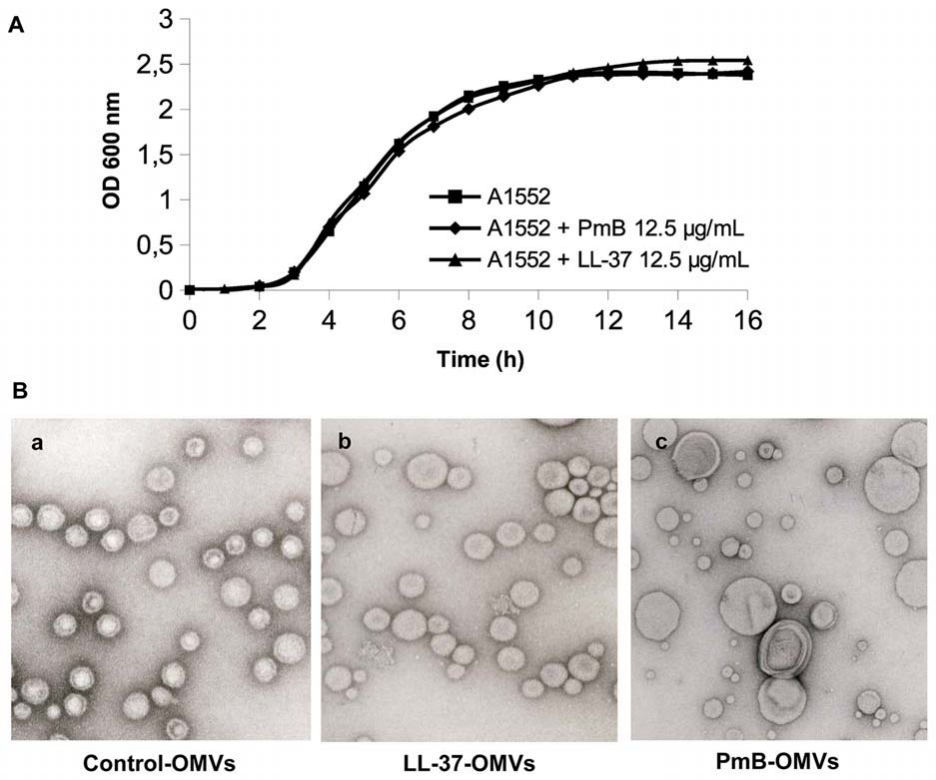
Sub-lethal AMP treatment and OMVs formation of *V. cholerae*. (A) Growth curves of A1552 in normal LB (square), LB supplemented with 12.5 µg/ml of PmB (diamond) and LB supplemented with 12.5 µg/ml of LL-37 (triangles). (B) Electron micrographs of OMVs from A1552 control cultures without AMPs (panel a) or cultures with a sub-lethal concentration of LL-37 (pannel b) or PmB (pannel c). Bars: 50 nm.

In order to analyse the effects of sub-lethal concentrations of AMPs on OMV production from the wild type *V. cholerae* strain A1552, bacteria were grown in presence of PmB or LL-37 as described in Materials and Methods. The bacteria were grown in presence of sub-lethal concentrations (¼ MIC) that did not alter the growth rate or yield (Fig. 1A). We isolated OMVs from cell-free supernatants of *V. cholerae* cultured with and without AMPs. Electron microscopy studies of the OMV samples revealed that OMVs from *V. cholerae* strain A1552 grown without AMPs (control-OMVs) were quite homogeneous in size, with a diameter of approximately 50 nm (Fig. 1B, panels a and b), whereas the OMVs isolated from *V. cholerae* grown with LL-37 (LL-37-OMVs) or PmB (PmB-OMVs) showed rather heterogeneous sizes with a diameter in the range of 10–100 nm (Fig. 1B, panel c). The majority of the PmB-OMVs were larger than those from the control-OMVs and the LL-37-OMVs (Fig. 1B, panels a, b and c). The overall amount of OMVs isolated from *V. cholerae* grown in presence and absence of AMPs were similar (Fig. 1B, panels a, b and c).

### Treatment of *V. cholerae* with AMPs modifies the protein profile but not the amount of OMVs produced

**Figure 2:**
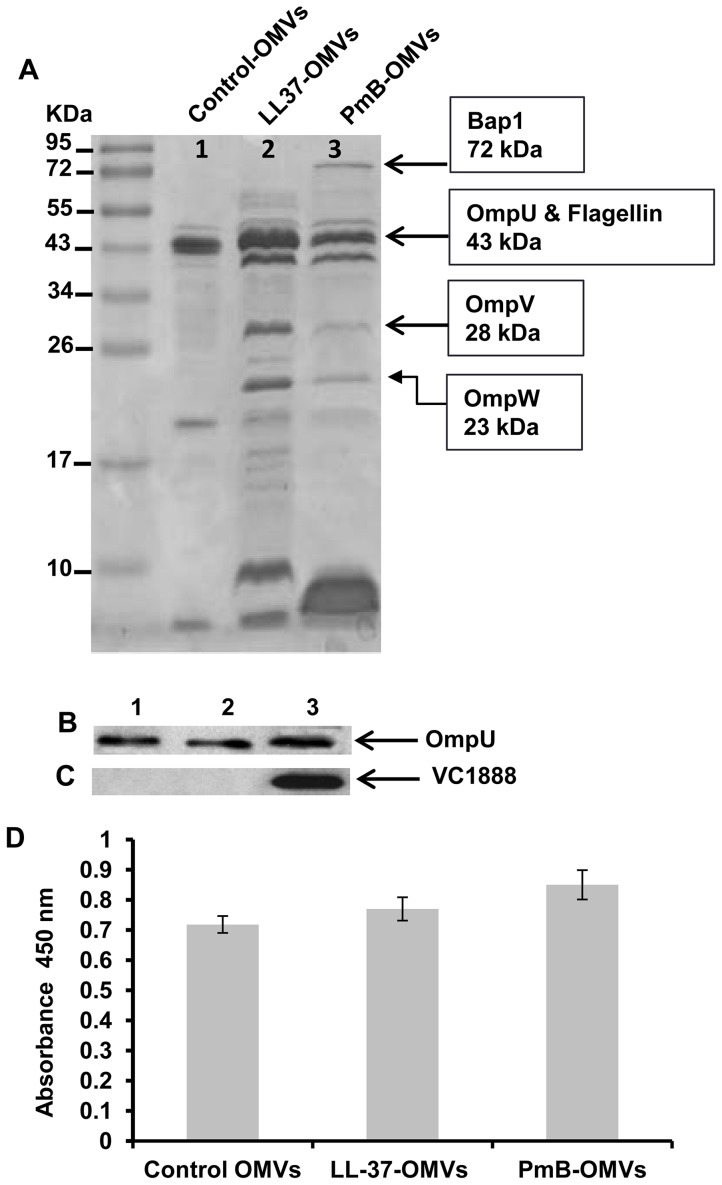
Analyses of OMVs from AMP treated *V. cholerae*. SDS-PAGE (A), immunoblot analyses using anti-OmpU (B) and anti-Bap1 antiserum (C) and quantification of OMV yield by analyzing LPS content by ELISA (D) of OMVs samples from control A1552 cultures (lane 1) or A1552 cultures with a sub-lethal concentration of LL-37 (lane 2) or PmB (lane 3).

In order to visualize the protein components of OMVs, we performed SDS-PAGE and examined whether growth in sub-lethal concentrations of AMPs influences the protein profile and amount of OMVs produced by *V. cholerae* A1552. The amount of OMVs released in presence and absence of AMPs were comparable, as shown by immunoblot analysis detecting the major outer membrane protein, OmpU, as a marker protein for *V. cholerae* OMVs (Fig. 2B) and by ELISA measurement of the LPS (Fig. 2D). These results were also in agreement with electron microscopic examinations (Fig. 1 A, B and C). However, a difference in the protein profile of the OMVs was found when the bacterial strain was grown with either PmB or LL-37 (Fig. 2A). Two extra bands observed at 28 kDa and 23 kDa (Fig. 2A, lanes 2 and 3) were identified by mass spectrometry (LC-MS/MS) as the outer membrane protein V (OmpV) and the outer membrane protein W (OmpW) of *V. cholerae*, respectively. The PmB- and LL-37-OMVs appear to have more OmpV and OmpW associated with them. Interestingly, in addition to increased levels of OmpV and OmpW, the PmB-OMVs showed an extra protein band at 72 kDa (Fig. 2A, lane 3). This band was identified by LC-MS/MS as VC1888, a biofilm-associated extracellular matrix protein, Bap1, of *V. cholerae*. We confirmed by Western blot analysis using polyclonal antiserum against Bap1 that this protein was associated with the OMVs of *V. cholerae* strain A1552 mainly when the bacteria were grown in the presence of a sub-lethal concentration of PmB (Fig. 2C, lane 3). Despite repeated LC-MS/MS analysis attempts, we were unable to obtain the identity of the additional protein bands in the molecular size range of 5–10 kDa that appeared in OMVs from bacteria grown in presence of LL-37 or PmB (Fig. 2A, lanes 2 and 3).

### OMVs from *V. cholerae* strain A1552 grown in presence of PmB can protect the bacteria against AMPs

**Figure 3:**
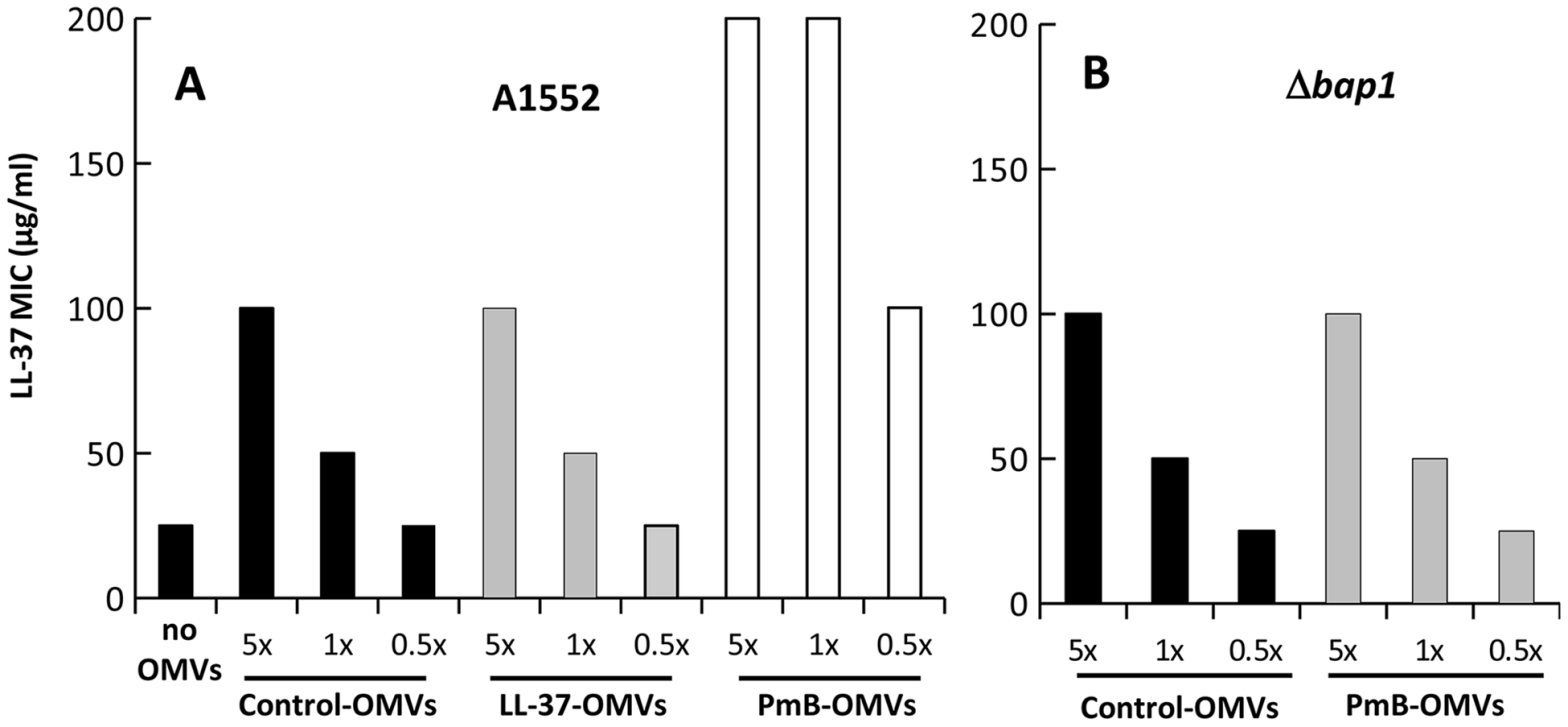
Altered AMP-protective effect of OMVs from PmB treated *V. cholerae*. Protective effect against LL-37 of OMVs from wild type A1552 grown with and without AMPs (A) and the Δ*bap1* mutant (B). Results are shown in µg/ml and represent the MIC values for LL-37 on A1552 measured after 1 h incubation of LL-37 with OMVs from A1552 grown in presence or absence of AMPs, followed by 16 h incubation with A1552 bacteria.

In order to analyse the role of OMVs in AMP resistance, OMVs were isolated from the wild type *V. cholerae* strain A1552 grown with sub-lethal concentrations of AMPs. The OMVs isolated from *V. cholerae* grown without AMPs were used as a control. To obtain a physiological concentration of OMVs, each OMV sample was suspended in 50 ml of PBS buffer, the volume of the bacterial supernatant used to isolate the OMV samples. This concentration is denoted as 1× in Fig. 3A and B. In this experiment, we used three different concentrations of OMV samples, i.e., physiological concentration (1×), 2 times diluted samples (0.5×), and 5 times concentrated samples (5×). The wild type *V. cholerae* strain A1552 was incubated together with AMPs in the presence of different OMVs preparations. The following combinations of the samples were used to measure minimum inhibitory concentration (MIC) values: A1552+OMVs from A1552 grown with PmB (PmB-OMVs)+PmB; A1552+PmB-OMVs+LL-37; A1552+OMVs from A1552 grown with LL-37 (LL-37-OMVs)+LL-37; A1552+LL-37-OMVs+PmB, A1552+OMVs from A1552 grown without AMPs (control-OMVs)+PmB; A1552+control-OMVs+PmB; and A1552+control-OMVs. As shown in Fig. 3A, when the bacterial strain was incubated with control-OMVs and LL-37-OMVs, there was no difference in MIC values for LL-37. However, the LL-37 MIC value was increased (from 50 to 200 µg/ml) when the bacterial strain was incubated with PmB-OMVs. We did not observe any difference in MIC values for PmB when the bacterial strain was incubated with control-OMVs, LL-37-OMVs, or PmB-OMVs (data not shown). These results indicate that OMVs from cultures in the presence of PmB can prevent inhibition of growth by LL-37 in a dose-dependent manner. Together, these results strongly support a role for OMVs in AMP cross-resistance in *V. cholerae*.

### Bap1 is implicated in resistance to AMPs

To investigate the role of Bap1 in AMP resistance, we constructed a Δ*bap1* mutation in the A1552 strain. Then we determined the MIC using the wild-type and the Δ*bap1* mutant challenged with either one AMP (PmB or LL-37) or a combination of both AMPs (PmB and LL-37). The results indicated that the Δ*bap1* mutant has the same level of resistance to AMPs as the wild-type with a MIC of 50 µg/ml when each AMP is used alone. However, when PmB and LL-37 were tested in combination with a fixed concentration of one AMP and an increasing concentration of the other AMP, we observed strong synergy between PmB and LL-37 for both the wild type and the Δ*bap1* strains. Indeed, the fractional inhibitory concentration (FIC) index was below 0.5 for both strains. Interestingly, the FIC index decreased from 0.10 to 0.02 upon mutating *bap1*, indicating that the synergistic effect of PmB and LL-37 was stronger for the Δ*bap1* mutant than for the A1552 wild-type. This result suggests that the Δ*bap1* mutant is more sensitive to the LL-37/PmB combination than the A1552 wild-type.

Moreover, in contrast with OMVs from the wild type strain A1552 grown in presence of PmB, OMVs from the Δ*bap1* mutant strain grown with PmB could not protect bacteria against AMPs. Indeed, the MIC for LL-37 was not altered after incubation with PmB-OMVs from the Δ*bap1* mutant (Fig. 3B), whereas the MIC after incubation with PmB-OMVs of wild-type strain A1552 was increased (Fig. 3A). Taken together, our results strongly support a role for the OMV-associated Bap1 protein as an effector of the cross-resistance between LL-37 and PmB in the *V. cholerae* O1 El Tor A1552 strain.

### Bap1 binds to OMVs through a major outer membrane protein, OmpT

**Figure 4:**
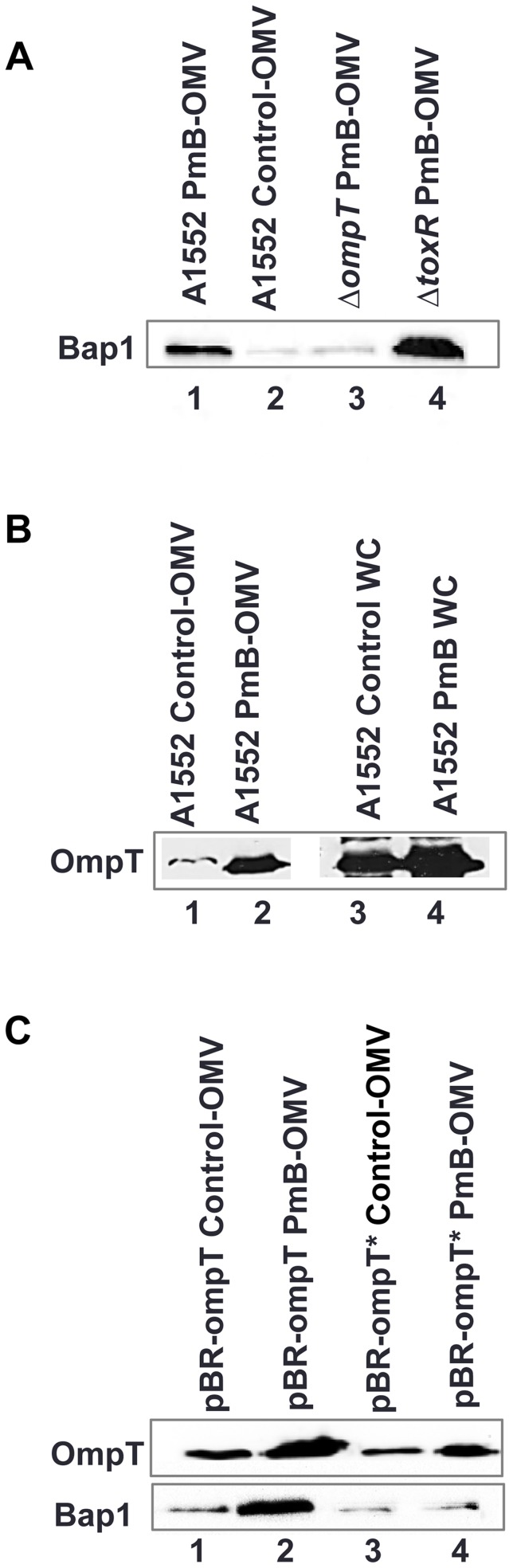
Immunoblot detection of OmpT and Bap1 in OMV samples and in total cell extracts from whole cell bacteria. (A) Detection of Bap1 in OMVs from A1552 grown with PmB (lane 1), OMVs from A1552 grown without OMVs(lane 2), OMVs from Δ*ompT* mutant grown with PmB (lane 3) and OMVs from Δ*toxR* mutant grown with PmB (lane 4). (B) Detection of OmpT in OMVs from A1552 grown without PmB (lane 1), OMVs from A1552 grown with PmB (lane 2), whole cell extract of A1552 grown without PmB (lane 3) and whole cell extract of A1552 grown with PmB (lane 4). (C) Detection of OmpT and Bap1 in control-OMVs isolated from Δ*ompT*/pBR-*ompT* (lane 1), in PmB-OMVs isolated from Δ*ompT*/pBR-*ompT* (lane 2), incontrol-OMVs from Δ*ompT*/pBR-ompT* (LDV mutant) (lane 3) and PmB-OMVs from Δ*ompT*/pBR-ompT* (LDV mutant) (lane 4).

Bioinformatics analyses (http://www.kegg.jp/ssdb-bin/ssdb_motif) show that the Bap1 protein contains four FG-GAP repeat domains, integrin-like structures that are important for ligand binding [19]. We investigated Bap1 binding to OmpT, a major outer membrane porin of *V. cholerae* containing an integrin-binding domain (LDV peptide). We isolated OMVs from the wild-type strain A1552; an *ompT* deletion mutant; and a deletion mutant of *toxR*, a negative regulator of *ompT* in the presence of sub-lethal concentrations of PmB. Western-blot analysis showed that PmB-OMVs from the wild type were associated with much higher quantities of Bap1 protein (Fig. 4A, lane 1) than those from control-OMVs (Fig. 4, lane 2). Interestingly, PmB-OMVs from A1552Δ*ompT* had significantly less associated Bap1 compared to wild type PmB-OMVs (compare Fig. 4 lanes 1 and 3). Moreover, PmB-OMVs from A1552 Δ*toxR* mutant bound more Bap1 protein than PmB-OMVs from wild-type A1552 (Fig. 4A lanes 1 and 4). In addition, the OmpT levels in OMVs and whole cell samples were increased upon PmB addition during A1552 growth as estimated by immunoblot analysis (compare Fig. 4B lanes 1 and 2; Fig. 4B lanes 3 and 4).

In order to decipher the binding mechanism between OmpT and the Bap1 protein, the amino acid residues LDV were deleted in OmpT by site-directed mutagenesis. We assessed the ability of Bap1 to bind the OmpT mutant by Western blot analysis. In presence of PmB, Bap1 was not able to bind to the OmpT protein from the Δ*ompT*/pBR-ompT* (LDV mutant) mutant to the same extent as to the wild type OmpT protein (Fig. 4C), suggesting a role for the LDV domain in Bap1 binding to OmpT on OMVs (Fig. 4C). Taken together, our results strongly support the hypothesis that Bap1 binds to OmpT on OMVs of *V. cholerae* A1552 through the LDV tripeptide integrin-binding domain of OmpT.

### OMVs from A1552 grown in presence of PmB can bind to but not degrade LL-37

**Figure 5:**
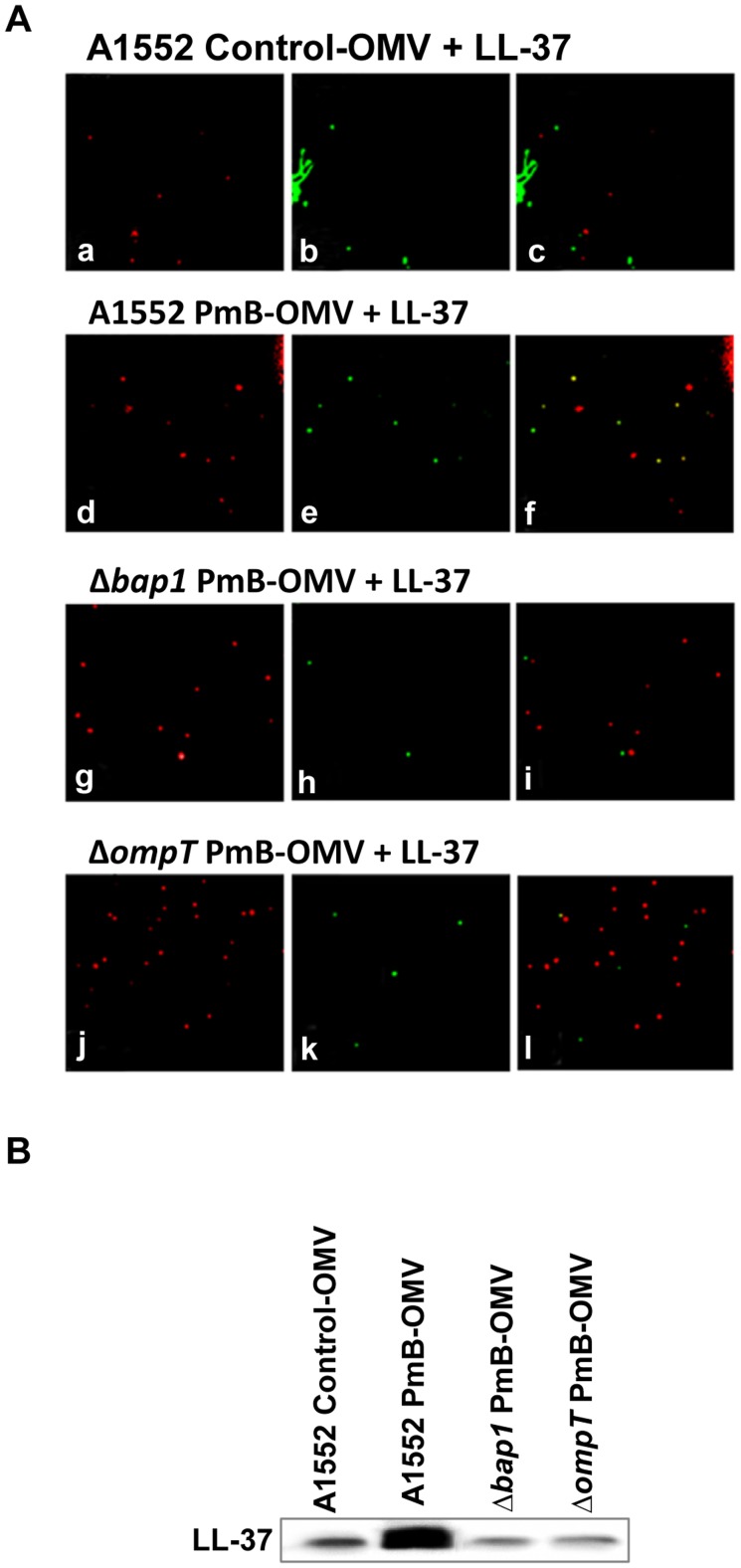
Detection of LL-37 binding to *V. cholerae* OMVs. (A) Fluorescence microscopy analysis of LL-37 binding to OMVs. Control-OMVs from A1552 grown without AMPs (a, b and c), A1552 OMVs grown with PmB (d, e and f), Δ*bap1* OMVs grown with PmB (g, h and i) and *ΔompT* OMVs grown with PmB (j, k, and l) were stained using PKH26 Red Fluorescent dye (a, d, g and j) and LL-37 was detected using anti-LL-37 and FITC-labelled (green) anti-rabbit antibodies (b, e, h and k). Merged images are shown in frames c, f, i and l. (B) Immunoblot quantification of LL-37 binding to control-OMVs from A1552, PmB-OMVs from A1552, PmB-OMVs from Δ*bap1* and PmB-OMVs from Δ*ompT*.

**Figure 6:**
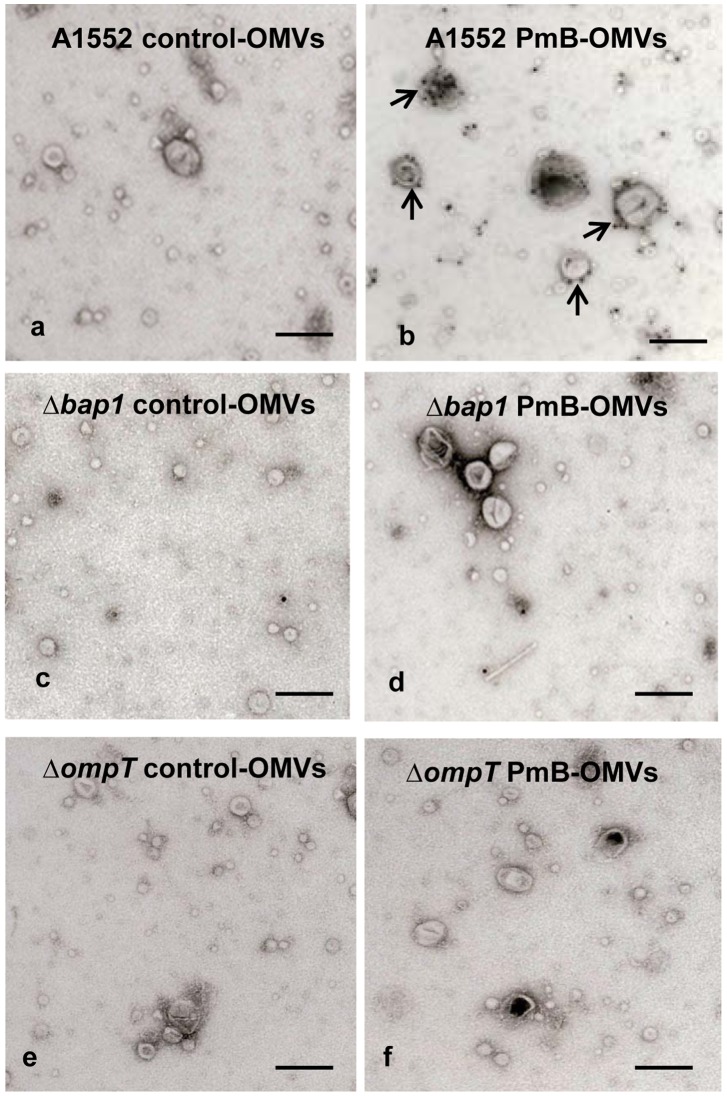
Immuno-gold labeling and electron microscopic analysis of LL-37 binding to OMVs. The following samples were analyzed: a) Control-OMVs from A1552 grown without AMPs, b) OMVs from A1552 grown with PmB, c) OMVs from the *Δbap1* mutant grown without PmB), d) OMVs from the *Δbap1* mutant grown with PmB, e) OMVs from the *ΔompT* mutant grown without PmB, f) OMVs from the *ΔompT* mutant grown with PmB. Arrows in panel b) indicate examples of gold labeling. Horizontal bars represent 100 nm.

In order to understand the mechanism of cross-resistance, we examined the interaction between LL-37 and PmB-OMVs from A1552. PKH26-labelled OMVs were incubated for 1 h with LL-37 and the AMP was detected by immunofluorescence using an anti-LL-37 antibody (Fig. 5A, a–l). Fluorescence microscopy revealed co-localisation of LL-37 and the OMVs from A1552 grown with PmB (Fig. 5A f), whereas no co-localisation was observed for the control OMVs of A1552 without AMPs (Fig. 5A c). We used the same method to examine the binding of OMVs from *V. cholerae* mutants to LL-37. PmB-OMVs from the Δ*bap1* mutant and from the Δ*ompT* mutant were assessed for binding of LL-37. No detectable LL-37 signal was observed in association with OMVs from these samples (Fig. 5A h and 5A k). The quantification by immunoblot analysis of LL-37 binding to OMVs from A1552, Δ*bap1*, and *ΔompT* mutants grown in presence or in absence of AMPs also showed that only A1552 wild-type PmB-OMVs displayed higher binding of LL-37 than other OMVs (Fig. 5B). To confirm the association of LL-37 on the surface of OMVs isolated from PmB treated bacteria, we performed immunogold labeling of LL-37 and electron microscopic analyses. As shown in Fig. 6, the gold particles were observed only on the surface of the OMVs isolated from wild type *V. cholerae* strain A1552 grown in presence of PmB (Fig. 6b) whereas there was no association of gold particles on the surfaces of OMVs isolated from *bap1* and *ompT* mutants grown with PmB or on control OMVs isolated from bacterial cultures without AMPs (Fig. 6a, c, d, e, f).

**Figure 7:**
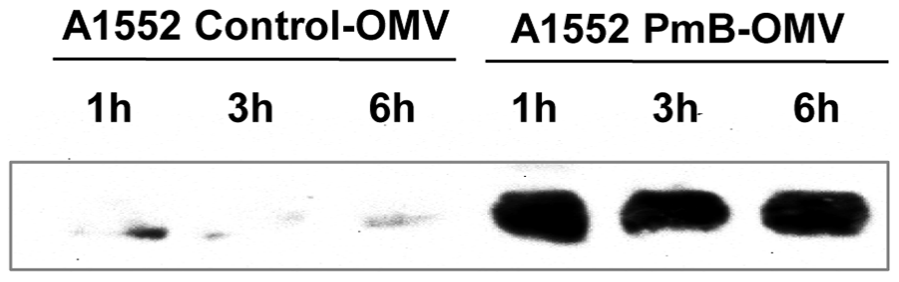
Effect of PmB treatment on LL-37 binding to *V. cholerae* OMVs. Immunoblot detection of LL-37 after 1 h, 3 h and 6 h of incubation with OMVs from A1552 grown without and with sub-lethal concentration of PmB.

We then asked whether LL-37 binding to A1552 PmB-OMVs would lead to degradation of LL-37. For this purpose, we performed time course incubations of vesicles and LL-37 and estimated subsequent AMP levels by immunoblot analysis using anti-LL-37 antiserum. Western blot analysis revealed no degradation of LL-37 after binding to PmB-OMVs (Fig. 7). This finding suggests that the OMVs from A1552 grown in presence of sub-lethal concentration of PmB can bind LL-37 with no subsequent degradation, and it is inferred from the above described studies that this binding requires the presence of the Bap1 protein.

In order to examine if free secreted Bap1 would protect the bacteria towards AMPs as OMVs associated Bap1 does, we used the Δ*ompT* mutant which secreted Bap1 at a level similar to that of wild type A1552 strain grown in both absence of PmB (Fig. S1; lanes 1 and 2) and presence of PmB (Fig. S1; lanes 4 and 5). However, a reduced amount of Bap1 protein was observed in association with the PmB-OMVs of the Δ*ompT* mutant in comparison with the amount of Bap1 protein associated with PmB-OMVs from wild type A1552 strain (Fig. 4A; lanes 1 and 3). Then we determined the MIC using the wild-type and the ΔompT and the Δ*bap1* mutants challenged with either one AMP (PmB or LL-37) or a combination of both AMPs (PmB and LL-37). The results indicated that the Δ*ompT* mutant has the same level of resistance to AMPs as the wild-type and the Δ*bap1* mutant (50 µg/ml), when each AMP is used alone. However, the FIC index calculated when AMPs are used in combination for the ΔompT and the Δ*bap1* mutants are the same (FIC index 0.02) and are lower than for the wild-type A1552 (FIC index 0.10). This result indicates that, when AMPs are used in combination, the Δ*ompT* mutant is more sensitive to AMPs than the wild-type A1552. Moreover, the Δ*ompT* and the Δ*bap1* mutant display the same sensitivity toward a combination of PmB and LL-37. This result strongly suggests that OMVs are important for the Bap1 mediated resistance to AMPs.

## Discussion

Adaptive resistance to antibiotics is well established in several bacterial species [20], [21]. Similarly, adaptive resistance to cationic antimicrobials including the polymyxin was first described decades ago [22], [23], although the specific mechanisms involved are only now being elucidated. Certain environmental factors lead to increased resistance to cationic peptides in *Pseudomonas aeruginosa*, including low Mg^2+^ concentrations [24], [25]; phosphate deprivation via the two-component regulatory system PhoPQ [26]; and reduction of the negative charge on LPS, which hinders the ability of polycationic lipopeptide polymyxins and antimicrobial peptides to mediate their self-promoted uptake across the outer membrane [27], [28]. Recently, Fernández et al. [24] identified the cationic peptide-mediated induction of LPS modification through the two-component system ParRS. In *V. cholerae*, a major outer membrane protein, OmpU, and an RND-family efflux system were shown to be involved in resistance to PmB and to a peptide derivate from the human bactericidal/permeability-increasing protein (BPI) [29], [30]. More recently, a role for the OMVs of *Escherichia coli* has been reported in AMP resistance [31].

We analysed the role of OMVs in AMP resistance of the *V. cholerae* O1 strain A1552. The bacterial strain was grown with PmB and examined for the release of OMVs from the bacterial cells. OMV production was not increased by incubating bacteria with PmB, as shown in our immunoblot studies using anti-OmpU antibody as an OMV marker, and by EM and ELISA analyses. Then we examined the morphology of OMVs isolated from bacterial cultures in the presence of PmB by electron microscopy and found that the OMVs prepared from the cultures with and without PmB did not appear different in the amount of vesicles *per se*. However, we observed larger OMVs when the bacteria were treated with a sub-lethal concentration of PmB in comparison with untreated samples. This finding differs from the earlier studies by Manning et al [31], who reported that PmB-treated *E. coli* bacterial cells released more OMVs into the culture supernatant although the OMVs did not appear morphologically different from untreated samples. This discrepancy might be due to the difference in AMP resistance between *E. coli* and *V. cholera*. *E. coli* strains are very sensitive to PmB treatment compared to *V. cholerae* strains. In addition, OMVs released from *V. cholerae* bacterial cells grown in the presence of sub-lethal concentration of PmB were associated with the extracellular matrix biofilm-associated protein Bap1 (VC1888). Due to the association of Bap1 on the surface of the vesicles or the increased levels of outer membrane OmpV and OmpW in the OMVs, the morphology of OMVs appeared to be altered.

Furthermore, we observed that the Bap1 protein can bind to the surface of the OMVs via OmpT when the OMVs were treated with PmB. By computational protein domain analysis (http://www.kegg.jp/ssdb-bin/view_sequence), an LDV domain was found at amino acid positions 262–264 of the OmpT protein (368 amino acids). We suggest that due to PmB treatment of the bacterial strain, the OmpT LDV residues become exposed during biogenesis on the surface of the OMVs, allowing for binding of the Bap1 protein and thereby promoting LL-37 binding to Bap1. Similar observation has been made by Mathur et al. who suggested that AMPs may promote a conformational change in mature OmpU of *V. cholerae* such that it exposes normally hidden C-terminal YDF motifs [30].

The Bap1 protein is also involved in rugose colony development and biofilm formation in *V. cholerae* [32], [33]. Bioinformatics analyses showed that the Bap1 protein contains four FG-GAP repeat domains and two VCBS domains. The FG-GAP repeats are found in the N terminus of integrin alpha chains, a region that is important for ligand binding and binding with proteins in the extracellular matrix or outer surface proteins of other cells [19], [34]. The approximately 100-residue VCBS domain is found as multiple (up to 35) copies in proteins from several species of *Vibrio, Colwellia*, *Bradyrhizobium*, and *Shewanella* (hence the name VCBS).

The function of VCBS domains is unclear although it has been suggested to be involved in cell adhesion (http://www.jcvi.org/cgi-bin/tigrfams/HmmReportPage.cgi?acc=TIGR01965).

In the present study, we showed that the *V. cholerae* mutant lacking Bap1 has significantly reduced binding efficiency of LL-37 to OMVs from *V. cholerae* grown with a sub-lethal concentration of PmB in comparison with OMVs from the wild type strain A1552 incubated with the same concentration of PmB. The reason might be that the Bap1 protein can act as an adhesin or in a ligand binding manner to promote binding to LL-37. In general, the membrane binding of AMPs may be driven by the electrostatic interactions between the positive peptide charges and the negative charges located in the phospholipid polar heads [35]. Gram-negative bacterial outer membranes are rich in acidic phospholipids and negatively charged LPS [36]. It is generally accepted that the highly cationic LL-37 interacts with these anionic bacterial outer membrane components by electrostatic forces [37], [38]. It is effective against both Gram-positive and Gram-negative bacteria where it binds to negatively charged targets in the bacterial cell wall and the cytoplasmic membrane whereupon it can kill the bacteria by disrupting membrane integrity. A recent study suggested that it also inhibits bacterial growth by affecting cell wall biogenesis [39]. Human LL-37 contains 11 positively charged residues (five arginines and six lysines), but also has five negatively charged amino acids (two aspartic acids and three glutamic acids), making its net charge +6. However, the estimated charge of the Bap1 protein monomer is −12.9 at pH 7.0 (http://www.scripps.edu/~cdputnam/protcalc.html), providing a possible explanation for the binding of the LL-37 peptide to the negatively charged target protein, the Bap1 protein, by an electrostatic interaction. The mechanism(s) of interaction between the LL-37 and the Bap1 protein is under investigation in our laboratory.

**Figure 8:**
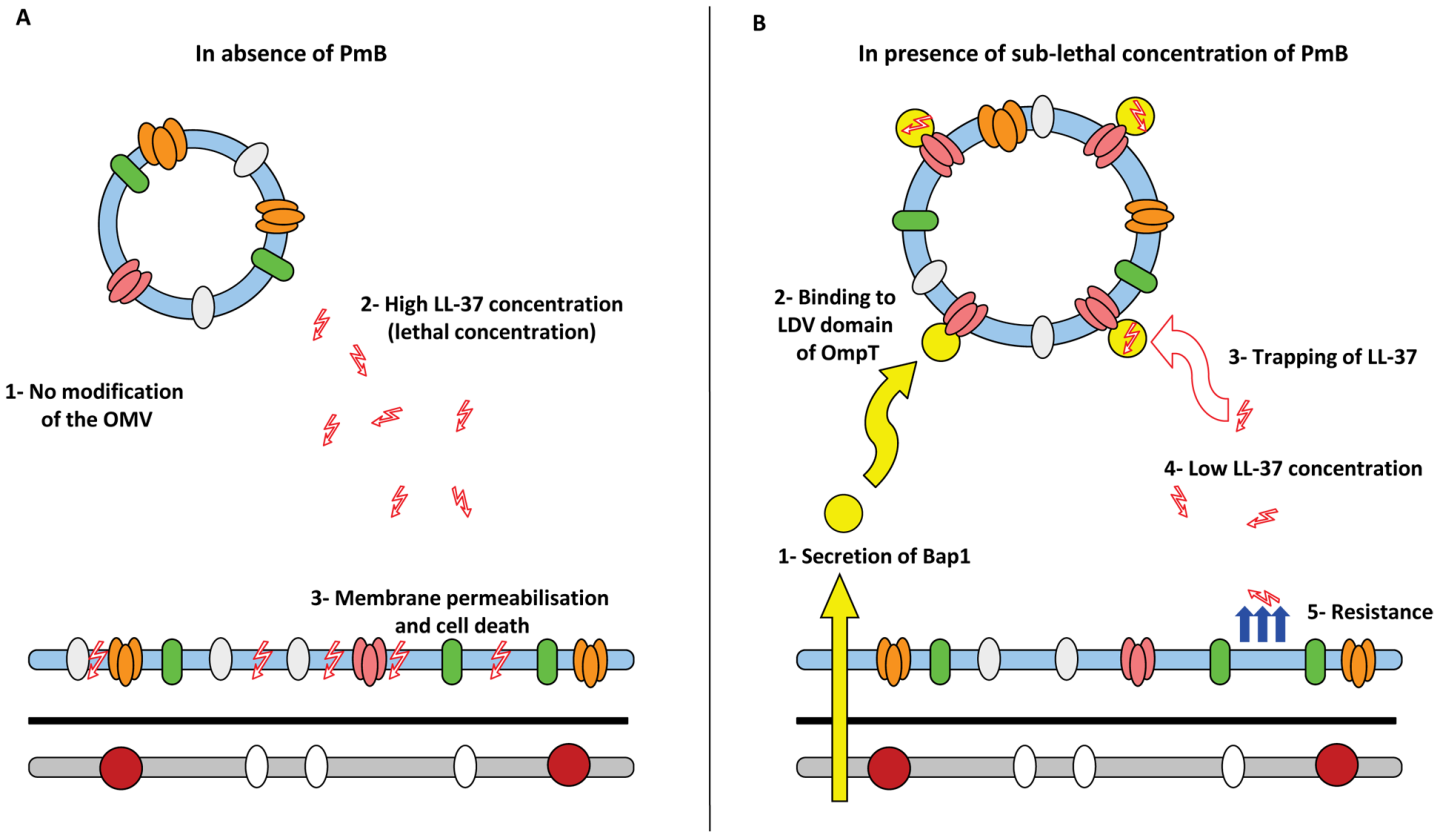
Model for the role of OMVs from *V. cholerae* grown with sub-lethal concentration of PmB in cross-resistance mechanism against LL-37 in *V. cholerae*. (A) In the absence of PmB, OMVs cannot protect A1552 against a lethal concentration of LL-37, leading to bacterial death. (B) Growing A1552 with a sub-lethal concentration of PmB can induce the release of OMVs able to bind Bap1 through the LDV domain of OmpT. Bap1 then serves as an adapter protein between LL-37 and the OmpT on the surface of the OMVs. The concentration of free LL-37 was thereby reduced to a sub-lethal concentration, leading to the apparent resistance and survival of *V. cholerae*. OM: outer membrane, PG: peptioglycan, IM: inner membrane.

We suggest that the Bap1 protein can serve as a scaffolding protein between the LL-37 peptide and the OmpT protein on the surface of OMVs isolated from *V. cholerae* grown with PmB. Due to the binding of LL-37 to Bap1-associated OMVs, the concentration of “free” LL-37 in the mixture is reduced to some extent and bacteria thereby become protected against LL-37. This protection might be applicable in the *in vivo* situation as the sub-lethal concentration of PmB can induce release of OMVs from *V. cholerae*, which can bind the Bap1 protein on the surface of the OMVs due to a conformational change in the OmpT protein. The Bap1-associated OMVs can bind to LL-37 and bacteria show cross-resistance against the LL-37 peptide (Fig. 8).

Regarding natural resistance against AMPs, it was described that resistance to AMPs develops at rather low frequencies. In earlier studies it was found that resistance of *Staphylococcus aureus* to protegrin, an AMP isolated from pigs, arose less frequently than even resistance to vancomycin [40]. Researchers aim to develop new types of antimicrobial therapy using AMPs that exhibit microbicidal and immuno-modulatory activity. However, as in the case of antibiotics, bacteria can evolve resistance to AMPs, risking the possibility that bacteria will also be able to resist the first arm of the human immune system. It has been suggested that selection for bacteria that evolved resistance to one AMP can confer some cross-resistance to a natural host-defense peptide [41]. Using experimental evolution, Habets and Brockhurst demonstrated that *S. aureus* rapidly evolved resistance to pexiganan, an AMP used for diabetic leg ulcer infections [42]. In some bacterial populations, experimentally evolved resistance to pexiganan provided *S. aureus* with cross-resistance to human-neutrophil-defensin-1, a key component of the innate immune response to infection. In our present study, we provide the first mechanistic evidence of cross-resistance to AMPs in bacteria using *V. cholerae* as a model organism. Our results should encourage further studies on the role and mechanisms of AMP cross-resistance in the intestinal lumen of the host, where both invading organisms and commensal organisms come into contact.

## Materials and Methods

### Bacterial strains and plasmids

The strains and plasmids used in the present work are described in Table 1 and the primers used are described in Table 2. Unless otherwise stated, the strains were grown at 37°C in either Luria-Bertani (LB) broth with vigorous shaking or on LB agar. When necessary, antibiotics were added at following concentrations: Carbenicillin (Cb)−100 µg ml^−1^ and Rifampicin (Rif)−100 µg ml^−1^.

**Table 1:** Strains and plasmids used in this study.

Strain/Plasmid	Description/Relevant characteristics	Reference/Source
*V. cholerae* strains		
A1552	O1 El Tor, Inaba, Rif^R^	Yildiz *et al.* 1998 [45]
A1552Δ*bap1*	Δ*bap1* derivative of A1552	This study
A1552Δ*toxR*	Δ*toxR* derivative of A1552	Song *et al.* 2010 [46]
A1552Δ*ompT*	Δ*ompT* derivative of A1552	Song *et al.* 2010 [46]
Plasmids		
pBAD18	arabinose inducible vector, Cb^R^	Guzman *et al.* 1995 [47]
pJET1.2	cloning vector, Cb^R^	Fermentas
pBR322	cloning vector, Cb^R^, Tc^R^	Bolvar *et al.* 1977 [48]
pBR-*ompT*	pBR322, *ompT* gene from A1552	This study
pBR-ompT*	pBR-*ompT*, LDV tripeptide substitution	This study

**Table 2:** Primers used in this study.

Primer name	Primer sequence (5′ to 3′)	Bold letters
DS1vc1888A	CGC**TCTAGA**TACAAGCCGGCATGCTTGA	*Xba*I site
DS2vc1888B	CCCATCCACTAAACTTAAACATTTCATGGCTTGACCTTCATC	
DS3vc1888C	TGTTTAAGTTTAGTGGATGGGAGTAAAGATACTTCTGCCAG	
DS4vc1888D	CGC**TCTAGA**AACTCATTAATCGCCACCAT	*Xba*I site
F-clone vc0930	GC**GAATTC**AGGTTGT TATTAGCAATCCGC	*Eco*RI site
R-clone vc0930	GC**TCTAGA**TTAGTAGACAAACTGGAAGCC	*Xba*I site
OmpT1up	CCG**AAGCTT**GGTAGCACAGATCAC	*Hind*III site
OmpT2do	CCG**GGATCC**TGAAAATGACAACAG	*Bam*HI site
OmpTmutup	CTTACTACAACGCAGAA**CATATG**GAAAATAACCCACTAGTG	*Nde*I site
OmpTmutdo	CACTAGTGGGTTATTTTC**CATATG**TTCTGCGTTGTAGTAAG	*Nde*I site

### AMPs susceptibility assays

MIC determination: Liquid growth inhibition assays (Hetru and Bulet, 1997) were performed in Poor Broth medium (1% Bactotryptone, pH 7.5). Growth (OD) was monitored spectrophotometrically at 600 nm for 16 h at 37°C using an Infinite 200 microplate reader (Tecan). MIC values are expressed as the lowest concentration that causes 100% growth inhibition (µg/ml).

Fractional inhibitory concentration (FIC) index: Serial dilutions of LL-37 were tested against a fixed concentration of half the MIC of PmB in 96-well plate. The same procedure was tested with a fixed concentration of LL-37 and a variable concentration of PmB. MIC was determined as described above. Results were expressed as the FIC index according to the following formula: FIC = (MIC of PmB, tested in combination)/(MIC of PmB, tested alone)+(MIC of LL-37, tested in combination)/(MIC of LL-37, tested alone). A FIC index of ≤0.5 indicates strong synergy (representing the equivalent of a fourfold decrease in the MIC of each compound tested), between 0.5–1.0 indicates synergy, ≥1 indicates that the antimicrobial activity of the two compounds is additive (a twofold decrease in the MIC of each compound tested),  = 2 indicates no effect and >2 indicates antagonism.

### OMV isolation

OMVs were isolated from the bacterial cultures as previously described (Wai et al. 2003). Briefly, bacteria were inoculated in a 200-ml culture flask containing LB and incubated for 16 h at 37°C with shaking. Tree different cultures were performed: A1552 in LB, A1552 in LB supplemented with one fourth of the MIC of PmB (12.5 µg/ml), and A1552 in LB supplemented with one fourth of the MIC of LL-37 (12.5 µg/ml). Bacterial cells were removed from culture fluid by centrifugation at 5000× *g* for 15 min. The supernatant was filtered through a 0.22-µm pore size PVDF membrane filter (Millipore). The cell-free supernatant was centrifuged at 100,000× *g* for 2 h at 4°C in a 45 Ti rotor (Beckman Instruments Inc.) to pellet the vesicles. The vesicles were suspended in 20 mM Tris-HCl (pH 8.0) or PBS.

### ELISA using anti-LPS antiserum for measurement of OMVs yield

The wells of microtiter plates were coated with OMVs isolated from PmB-treated or LL-37-treated or untreated wild type *V. cholerae* strain A1552 in a coating buffer (0.2 M sodium carbonate, pH 9.4). The plate was incubated at 4°C overnight. The plate was then washed three times with washing buffer (0.01% Tween 20 v/v in PBS). To each well, 100 µl of rabbit polyclonal antibody raised against LPS of El Tor Inaba *V. cholerae* (Difco), at a dilution of 1 in 1000 in washing buffer, was added. The plate was incubated for 1 h at room temperature. Subsequently, the plate was washed three times with washing buffer and 100 µl of anti-rabbit IgG-HRP conjugate diluted 1 in 3000 in washing buffer was added to each well and the plate was incubated 1 h at room temperature. The plate was washed three times and the enzymatic reaction was initiated by the addition of 100 µl of 3,3′, 5,5′- tetramethylbenzidine (TMB) substrate (Thermo Scientific) to each well containing peroxide. The plate was incubated at room temperature for 30 min before 100 µl of stop solution (0.16 M sulfuric acid) was added to each well. The absorbance was measured at 450 nm.

### Immunogold labelling and electron microscopic analysis

For immunoelectron microscopy, a colloidal gold probe (Wako Pure Chemical Industries Ltd., Osaka, Japan) was used to label the reaction sites of anti-LL-37 serum in the specimens of OMVs from *V. cholerae* wild type strain A1552 and its derivatives. To label the specimens, a 50 µl sample (ca 3 µg protein) of the OMVs preparation was incubated with LL-37 (1 mg/ml) for 30 min at 37°C before washing three times with phosphate-buffered saline (PBS) to remove unbound LL-37. This OMVs sample was then treated with LL-37 antiserum, appropriately diluted in PBS for 30 min at 37°C and subsequently separated from the serum by centrifugation at 100,000× g for 2 h at 4°C. After three times washing with PBS, the OMVs samples were mixed with a suspension of the colloidal gold probe, and the mixture was kept at room temperature for 30 min. After washing with PBS to remove unbound gold particles, the OMVs samples were negatively stained with 0.1% uranyl acetate on carbon coated Formvar grids and examined under the electron microscope. Micrographs were taken with a JEOL 2000EX electron microscope (JEOL Co., Ltd., Akishima, Japan) operated at an accelerating voltage of 100 kV.

### Construction of the *bap1* mutant

The *bap1* deletion mutant was constructed using procedures that have been described previously [43], [44]. The oligonucleotide primers used are: DS1vc1888A,DS2vc1888B,DS3vc1888C, and DS4vc1888D.

### Anti-RbmC polyclonal antiserum preparation

The *rbmC* gene was amplified by PCR using the primers F- clone vc0930 and R- clone vc0930 and cloned into pBAD18 using *Eco*RI and *Xba*I restriction enzyme sites. The expression of RbmC was induced using 0.02% of arabinose. The band containing the RbmC protein was excised from an SDS polyacrylamide gel and subsequently eluted from the gel slice. Using the eluted RbmC protein as antigen for immunization, polyclonal rabbit antiserum was produced by AgriSera AB, Sweden. Bap1, which has 53.7% amino acid sequence identity with RbmC, was found to cross react with our RbmC antiserum (see Fig. S1 and S2).

### Construction of the OmpT LVD mutant

A fragment of the *ompT* gene and promoter region with two CRP binding sites was PCR amplified from chromosomal DNA isolated from strain A1552 using primers OmpT1up and OmpT2do, which introduced additional *Hind*III or *BamH*I restriction endonuclease recognition sites at the ends. The PCR amplification was performed using Velocity DNA Polymerase (Bioline) according to the manufacturer's manual. The PCR product was cloned into pJET1.2 for sequencing and amplification purposes. A correct plasmid construct was digested with *BamH*I and *Hind*III and the gel-purified *ompT* fragment was cloned into *BamH*I and *Hind*III digested pBR322, resulting in plasmid pBR-*ompT*. pBR-ompT* was constructed from 300 ng pBR-*ompT* DNA using the QuikChange® Multi Site-Directed Mutagenesis Kit, Stratagene, according to the manufacturer's manual. The primers used, *ompT*mutup and *ompT*mutdo, create an *Nde*I site instead of the bases coding for the amino acid sequence LVD in the OmpT coding sequence.

### SDS-PAGE and Western-blotting analysis

The isolated OMVs were subjected to polyacrylamide gel electrophoresis using the method described by Laemmli (1970) and then blotted onto a PVDF membrane. Proteins were identified using anti-OmpU polyclonal antiserum, anti-Bap1 (Anti-RbmC) polyclonal antiserum or anti-OmpT polyclonal antiserum. Anti-rabbit horseradish peroxidase-conjugated antibody preparation (Dako) was used as a secondary antiserum at a final dilution of 1∶20,000. The ECL^+^ chemiluminescence system (GE Healthcare) was used to detect chemiluminescence, which was recorded using a Fluor-S MultiImager (BioRad) and by autoradiography.

### OMV protection assay

OMVs were diluted in order to reach physiological concentration (1×) [adjusted to the volume of the initial bacterial culture and defined as the concentration of OMVs obtained from a 16-h LB culture at 37°C] or five times the physiological concentration (5×), or half the physiological concentration (0.5×). OMVs at concentrations of 0.5×, 1× and 5× were then incubated with either LL-37 or PmB and A1152 bacteria for 16 h at 37°C and MIC was determined as previously described.

### LL-37 - OMV binding assay

OMVs (10 µL) were incubated with LL-37 (1 mg/ml) in a final volume of 100 µL for 1 h at 37°C. After centrifugation at 14,000 *g* for 30 min, OMVs were stained using the PKH26 Red Fluorescent Cell Linker Kit (Sigma) diluted 1∶500 and incubated for 5 min at room temperature before centrifugation at 18,000 *g* for 30 min. Anti-LL-37 polyclonal antibody (Innovagen) at a dilution of 1∶5000 was then added for 1 h before washing by addition of 200 µL of PBS and centrifugation at 18,000 *g* for 30 min. FITC-labelled anti-rabbit (Innovagen) was used as secondary antiserum at a final dilution of 1∶10,000 for 1 h. OMVs were then washed three times by centrifugation at 18,000 *g* for 30 min before fluorescence microscopy or immuno-detection analysis.

### LL-37 degradation assay

OMVs (10 µl) were incubated with LL-37 (100 µg/ml) in a final volume of 100 µL for 1 h, 3 h and 6 h at 37°C. After three PBS-washing steps and centrifugations at 18,000 *g* for 30 min, 15 µL were submitted to an 18% acrylamide gel and blotted onto a PVDF membrane. LL-37 degradation was monitored using a polyclonal anti-LL-37 antibody (Innovagen) at a final dilution of 1∶5000. Anti-rabbit horseradish peroxidase-conjugate (Dako) was used as a secondary antiserum and detection was performed as described above in in the section “SDS-PAGE and Western-blotting analysis”.

## Supporting Information

Figure S1 Immunoblot analysis of the secretion of Bap1 in the supernatants before OMVs isolation from cultures of A1552, Δ*ompT* and Δ*bap1* grown with and without PmB.
(TIF)

Figure S2 Immunoblot detection of Bap1 and RmbC using anti-RbmC anti-rabbit polyclonal antiserum in supernatants before OMVs isolation and in association with OMVs from A1552, Δ*bap1* and Δ*rbmC* grown in presence of PmB.
(TIF)
