# Supplementary figures and images for: Role of the Vibrio cholerae Matrix Protein Bap1 in Cross-Resistance to Antimicrobial Peptides
Source: PLoS Pathog. 2013 Oct 3;9(10):e1003620. doi: 10.1371/journal.ppat.1003620 (PMC3789753; doi:10.1371/journal.ppat.1003620)

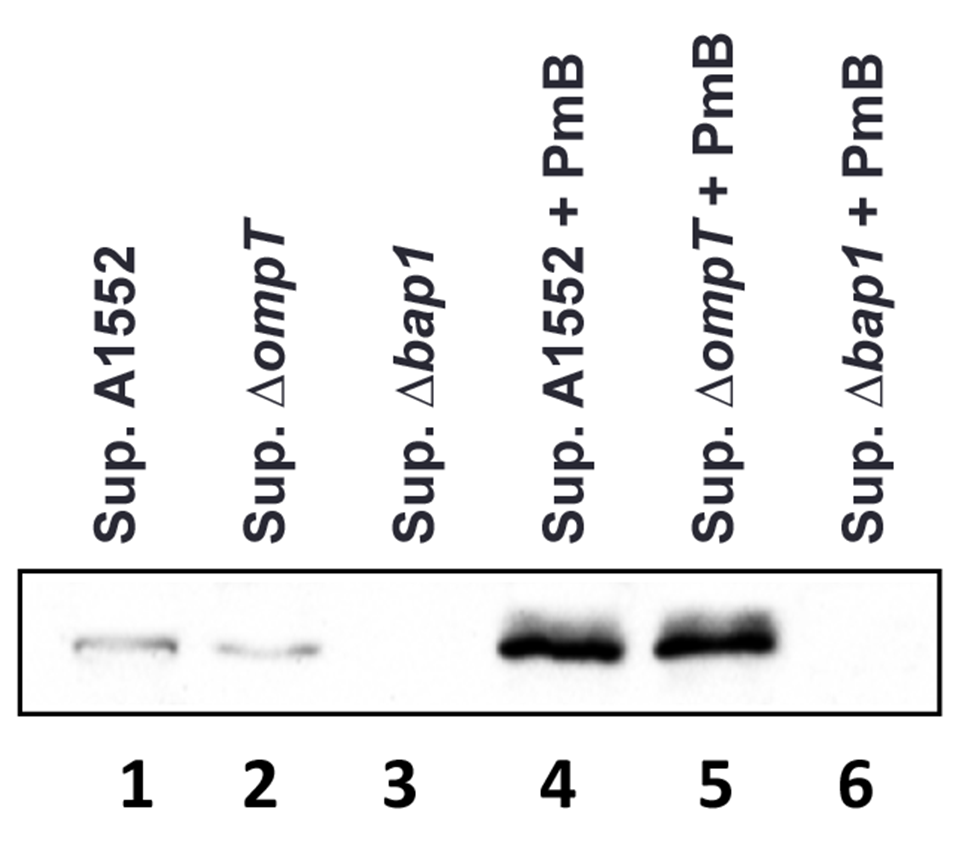

Supplement: Figure S1 — Immunoblot analysis of the secretion of Bap1 in the supernatants before OMVs isolation from cultures of A1552, ΔompT and Δbap1 grown with and without PmB. (TIF) [file ppat.1003620.s001.tif]

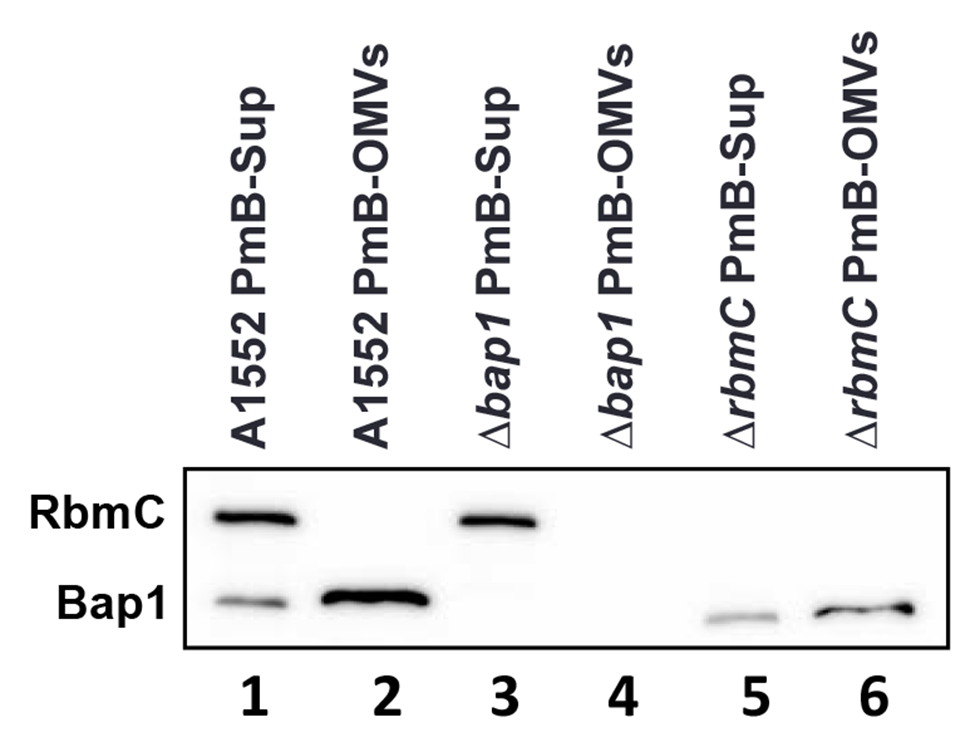

Supplement: Figure S2 — Immunoblot detection of Bap1 and RmbC using anti-RbmC anti-rabbit polyclonal antiserum in supernatants before OMVs isolation and in association with OMVs from A1552, Δbap1 and ΔrbmC grown in presence of PmB. (TIF) [file ppat.1003620.s002.tif]
